# A computational study of crimping and expansion of bioresorbable polymeric stents

**DOI:** 10.1007/s11043-017-9371-y

**Published:** 2017-10-30

**Authors:** T. Y. Qiu, M. Song, L. G. Zhao

**Affiliations:** 1 Wolfson School of Mechanical, Electrical and Manufacturing Engineering, Loughborough University, Loughborough, LE11 3TU UK; 2 Department of Materials, Loughborough University, Loughborough, LE11 3TU UK

**Keywords:** Bioresorbable polymeric stents, Finite element, Crimping, Expansion, U-bends, Recoiling, Rate effect

## Abstract

This paper studied the mechanical performance of four bioresorbable PLLA stents, i.e., Absorb, Elixir, Igaki–Tamai and RevaMedical, during crimping and expansion using the finite element method. Abaqus CAE was used to create the geometrical models for the four stents. A tri-folded balloon was created using NX software. For the stents, elastic–plastic behaviour was used, with hardening implemented by considering the increase of yield stress with the plastic strain. The tri-folded balloon was treated as linear elastic. To simulate the crimping of stents, a set of 12 rigid plates were generated around the stents with a radially enforced displacement. During crimping, the stents were compressed from a diameter of 3 mm to 1.2 mm, with the maximum stress developed at both inner and outer sides of the U-bends. During expansion, the stent inner diameter increased to 3 mm at the peak pressure and then recoiled to different final diameters after balloon deflation due to different stent designs. The maximum stress was found again at the U-bends of stents. Diameter change, recoiling effect and radial strength/stiffness were also compared for the four stents to assess the effect of design variation on stent performance. The effect of loading rate on stent deformation was also simulated by considering the time-dependent plastic behaviour of polymeric material.

## Introduction

Bioresorbable stents have been developed to provide short-term lumen support in plaque-blocked arteries and then disappear within a specific time period, which reduces the occurrence of thrombosis, in-stent restenosis and inflammation that usually appear after the deployment of permanent metallic stents (Garcia-Garcia et al. 2014). Bioresorbable stents are usually made of biodegradable polymer or magnesium alloy. The concept of bioresorbable stent can be dated back to early 1990s when Tamai et al. (2000) reported the first success of a fully degradable stent implanted in human. Since then, a variety of devices have been developed for preclinical and clinical evaluations, such as the Igaki–Tamai stent (Kyoto Medical Planning Co., Ltd., Kyoto, Japan), the Absorb (Abbott Vascular, Santa Monica, CA, USA), Elixir DESolve (Elixir Medical Corp., Sunnyvale, CA, USA) and REVA ReZolve (Reva Medical Inc., San Diego, CA, USA).

Many clinical studies have been carried out to assess the performances of bioresorbable polymeric stents. The Igaki–Tamai stents, the first fully biodegradable coronary stents made of Poly (L-Lactic acid) (PLLA), were implanted in 50 patients to evaluate the long-term ($>10$ years) clinical outcome. Results showed that the major adverse cardiac event rate was similar to that of bare metal stent, and low scaffold thrombosis rate (without stent recoil and vessel remodelling) was found in the follow-up trial, which suggested the long-term safety of the stents (Nishio et al. 2012). The Absorb stents, also made of PLLA, were firstly applied in the treatment of de novo coronary artery lesions for 30 patients, and 2-year follow-up outcomes suggested that the stent was fully absorbed over the period and also clinically safe with no cardiac deaths or stent thrombosis (Serruys et al. 2009). Recently, a 12-month clinical outcome has been published to present the mid-term performance of the Absorb stent in the treatment of focal tibial and distal popliteal lesions, demonstrating that the Absorb stent was safe and free from target lesion revascularisation (Varcoe et al. 2016). The novel PLLA DESolve novolimus-eluting bioresorbable coronary scaffold (Elixir) underwent a 2-year clinical trial in the treatment of single de novo coronary lesions for 126 patients, and the outcomes showed that early luminal growth was found at 6 months and efficacy and safety of the stents were sustained through 2 years (Abizaid et al. 2016).

Over the last two decades, great efforts have been made to study mechanical performance of bioresorbable polymeric stents using experimental methods. For instance, Grabow et al. (2007) manufactured novel slotted tube stents (made of PLLA and PLLA/PCL/TEC) and studied their mechanical properties for peripheral vascular applications. They found that pure PLLA stent had recoil (2.4%) and collapse pressure (0.67–1.3 bar) comparable to commercial peripheral metallic stent (recoil values of 2.5–4.8%, collapse pressures of 0.8–1.2 bar), due to the adequate mechanical properties of pure PLLA. Welch et al. (2009) investigated the influence of thermal treatment on the mechanical properties of coil-within-coil PLLA fibre stent. The results indicated that thermal treatment changed the reorientation and realignment of the fibre crystalline structure, and thereby improved the fibre stress-strain behaviour and expansive properties of the PLA fibre stent. In 2013, they also fabricated double-opposed helical stents (made of PLLA) and characterised their mechanical properties as a function of stent size and design. The results showed that the mechanical behaviour of stent was affected by stent design, such as the number of coils within the double helical design, and winding in longitudinal directions (Welch et al. 2013).

In addition, the finite element method has also been used to study the mechanical behaviour of stent by simulating deployment of stent. Migliavacca et al. (2005) carried out computational study of the free expansion of Cordis BX Velocity stent, and results showed a good match with the experimental studies in terms of radial expansion and elastic recoil. Gervaso et al. (2008) simulated metallic stent expansion by using three different inflation procedures: (i) imposing a uniform pressure on stent inner surface, (ii) expanding a rigid cylindrical surface by displacement control and (iii) modelling a polymeric deformable balloon. The three procedures gave similar stress distribution on the stent struts, but significantly different deformed shapes. De Beule et al. (2008) evaluated the effect of balloon folding on the free expansion behaviour of Cypher stent. Results suggested that the mechanical behaviour of tri-folded balloon-expandable stent showed the best agreement with both manufacturer’s data and experimental measurements. Schiavone et al. (2014) conducted a comparative study of the mechanical performances for four metallic stents (e.g. Palmaz–Schatz, Cypher, Xience and Endeavor stents), and reported that stent design is one of the major factors controlling the stent performance.

As discussed above, most computational studies focused on metallic stents. Computational analysis of bioresorbable polymeric stents, particularly PLLA stent, is very limited. Existing research is largely limited to the Absorb stent or simplified tubular PLLA stent. For example, Debusschere et al. (2015) examined the effect of balloon deployment rate on the expansion behaviour of Absorb stent by using implicit finite element solver, and results suggested that the stepwise balloon inflation method reduced the high stress level on expanded stent. Pauck and Reddy (2015) studied the radial strength, elastic recoil and radial stiffness of PLLA coronary artery stent using finite element method. Results revealed that material modulus is an important parameter in determining the mechanics of polymeric stent, and strut thickness and width also affected the mechanical behaviour of PLLA stent. Wang et al. (2017) studied the mechanical performance of unit Absorb stent by computational method. They revealed that the stress distribution, the stiffness and strength of stent were different when they were crimped and expanded to different diameters. As such, there is a research gap in the computational investigation of the mechanical behaviour of bioresorbable polymeric stents with different designs. Also, the time-dependent behaviour of polymeric materials was seldom accounted for in computer simulations of stent performance.

This paper aims to conduct computational investigation of the mechanical performances for four bioresorbable polymeric stents (Absorb stent, Elixir stent, Igaki–Tamai stent and RevaMedical stent) during crimping and expansion processes. A comparative study for these four polymeric stents was presented, in terms of stress distribution, diameter change against pressure, recoiling effect as well as radial stiffness and strength. Furthermore, stent expansion was also simulated to study the effects of crimping-caused residual stress, as well as loading rate, on the behaviour of bioresorbable polymeric stents.

## Methodology

### Finite element models

Four different bioresorbable polymeric stent designs were considered in this study, and they are the Absorb stent, Elixir stent, Igaki–Tamai stent and RevaMedical stent. The Absorb and Elixir stents have obtained CE mark (“CE” means European Conformity, and “CE mark” is a symbol of free marketability in the European Economic Area) to treat coronary artery disease, while the Igaki–Tamai stent obtained the CE mark to treat peripheral artery disease. These three stents are made of Poly (lactic-L-acid) (PLLA) (Wiebe et al. 2014). The RevaMedical stent is made of polycarbonate, and has been undergoing clinical evaluation (Garcia-Garcia et al. 2014).

**Fig. 1:**
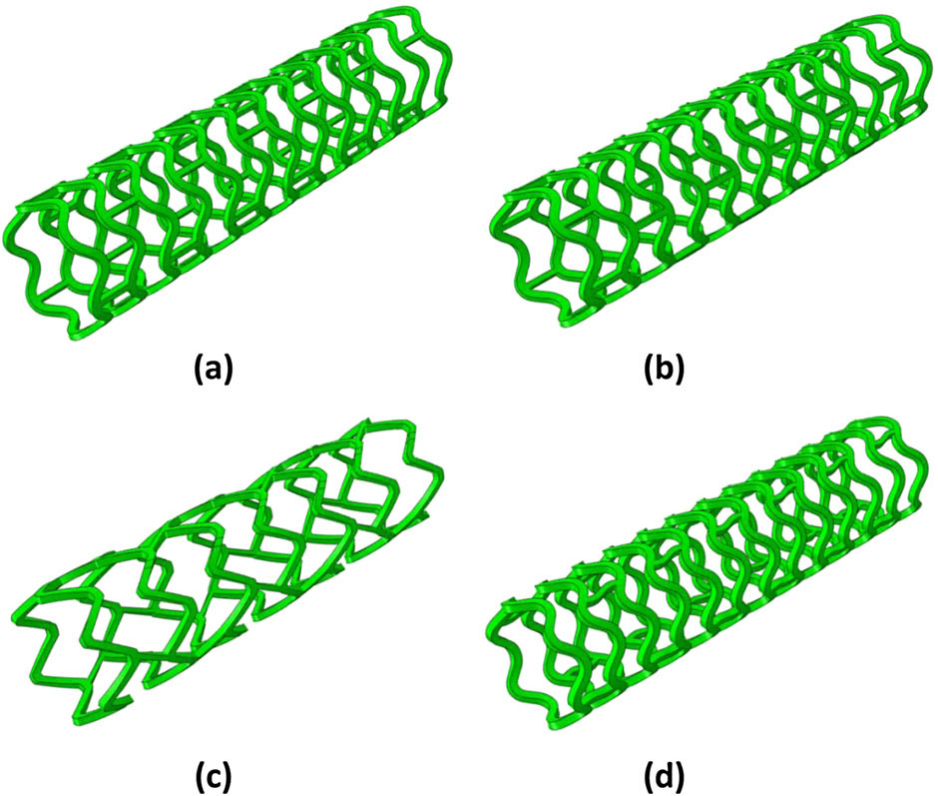
3D models for (**a**) Absorb, (**b**) Elixir, (**c**) Igaki–Tamai and (**d**) RevaMedical stents

Abaqus 6.14 CAE was used to build the 3D finite element models for these four stents, as shown in Fig. 1. C3D8R element was used to mesh the stent models. NX 8.5 (Siemens PLM Software, UK) was used to create the tri-folded balloon, and then imported in Abaqus CAE. The length of the tri-folded balloon is 16 mm for the middle part, and the fully inflated diameter is about 3 mm. The 4-node quadrilateral membrane element was chosen to mesh the balloon, and the balloon model has 12012 elements in total.

### Material models

**Fig. 2:**
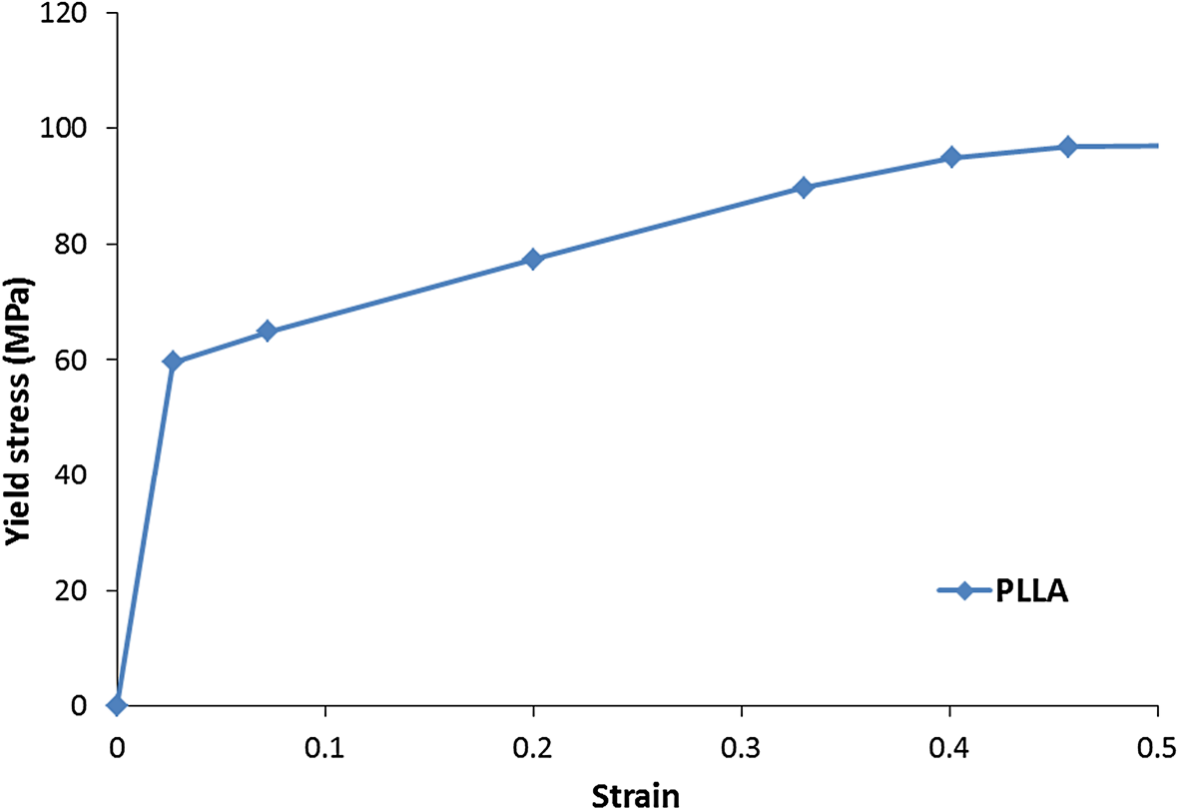
Stress-strain behaviour for PLLA used in simulations

Bioresorbable stents are made of biodegradable Poly (lactic-L-acid) (PLLA). The mechanical property for PLLA was found in Pauck and Reddy (2015), who obtained stress-strain curve through performing uniaxial tensile tests (Fig. 2). The density of material is $1.4\times 10^{-6}$, Young’s modulus is 2200 MPa and Poisson’s ratio is 0.3. In simulation, plastic behaviour was described by providing the yield stress as a function of plastic strain extracted from the stress-strain curve in Fig. 2. Tri-folded balloon was defined as an isotropic and linear-elastic material, with Young’s modulus of 900 MPa and Poisson’s ratio of 0.3 (Gervaso et al. 2008).

The material model for the stent follows the classical time-independent plasticity model, which uses von Mises yield surfaces with isotropic hardening. The model is intended for applications involving plastic deformation under monotonic loading condition such as crash analyses, metal forming and general collapse studies. Plasticity models that include kinematic hardening are more suitable for cases involving cyclic loading. In this paper, we are interested in monotonic deformation of stent during crimping and expansion, for which isotropic hardening is sufficient (for fatigue analysis such as deformation under pulsatile blood pressure, both isotropic and kinematic hardening are recommended). The model is very well known in the mechanics community and also well documented in ABAQUS (2016). To help understand the model, some of the key equations are given below.

Essentially, the strain tensor $\boldsymbol{\varepsilon }$ has two parts, i.e., an elastic part $\boldsymbol{\varepsilon}_{e}$ and an inelastic part $\boldsymbol{\varepsilon}_{p}$:

1 $$ \boldsymbol{\varepsilon} = \boldsymbol{\varepsilon}_{e} + \boldsymbol{\varepsilon}_{p}. $$

The elastic strain $\boldsymbol{\varepsilon}_{e}$ obeys Hook’s law, as follows:

2 $$ \boldsymbol{\varepsilon}_{e} = \frac{1 + \nu }{E}\boldsymbol{\sigma} - \frac{\nu }{E} ( \operatorname{tr} \boldsymbol{\sigma} ) \boldsymbol{I}, $$

where $E$ and $v$ are the Young’s modulus and the Poisson’s ratio of the material, $\boldsymbol{\sigma }$ and $\boldsymbol{I}$ stress tensor and the unit tensor of rank two, respectively, and $\operatorname{tr}$ the trace.

According to the von Mises yield criterion, the yield function $f$ is defined as

3 $$ f (\boldsymbol{ \sigma} ,H,k ) = J ( \boldsymbol{\sigma} ) - H - k \le 0, $$

where $H$ is the isotropic hardening variable and $k$ is the initial value of the radius of the yield surface. $J$ denotes the von Mises distance in the deviatoric stress space

4 $$ J ( \boldsymbol{\sigma} ) = \sqrt{\frac{3}{2}\boldsymbol{\sigma} ':\boldsymbol{\sigma} '}, $$

where $\boldsymbol{\sigma }'$ is the deviator of $\boldsymbol{\sigma }$ and “:” represents the inner product of two tensors. Plastic flow occurs under the condition $f = 0$ and $\frac{\partial f}{ \partial \boldsymbol{\sigma} }:\dot{\boldsymbol{\sigma} } > 0$.

Using the classical normality rule, the plastic strain becomes

5 $$ \boldsymbol{\varepsilon}_{p} = \lambda \frac{\partial f}{\partial \boldsymbol{\sigma} }, $$

where the plastic strain multiplier $\lambda $ is identical to accumulated plastic strain $p$, i.e., $\lambda = p$.

The evolution of the isotropic hardening is described as a function of plastic straining:

6 $$ H = f ( p ), $$

where $p$ is the accumulated plastic strain. In ABAQUS, the isotropic hardening was considered by providing the yield stress as a tabular function of plastic strain.

### Stent crimping procedure

**Fig. 3:**
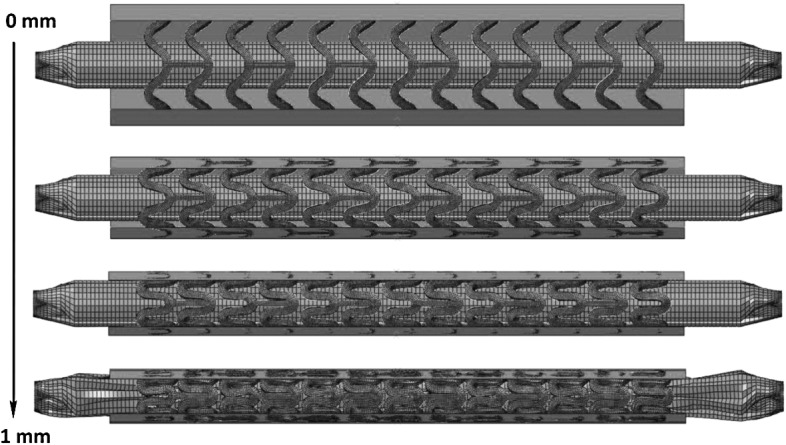
Procedure of stent crimping

In this study, 12 rigid plates were used to crimp the stent onto the balloon controlled by enforced displacement loading, as shown in Fig. 3. Both ends of balloon were fully constrained during the simulation and hard contact between stent outer surface and rigid plates was specified with a friction coefficient of 0.8. This ensured that there was no slip between the stent and the rigid plates during crimping. We tried a few other values (as there was no physical measurement in the literature) and found that this value (or a higher one) was fit for the purpose. A similar value was also used in the work of Schiavone et al. (2016, 2017). An additional spring-back step was also included by releasing the 12 rigid plates after stent crimping, which allowed for a recovery of elastic deformation of the crimped stent.

### Stent expansion procedure

For stent expansion, pressure was applied on the inner surface of tri-folded balloon to inflate the stent. The pressure increased from 0 to 1.4 MPa in 0.1 s. The simulation is carried out using ABAQUS explicit which requires a time. The use of 0.1 s is close to the actual inflation time in surgery. We also tried different times (0.01 s, 0.1 s and 1 s) to simulate the balloon inflation procedure, and no significant difference was observed (von Miss stress distribution, stent diameter change and recoiling effect). Also, the choice of 0.1 s helped to achieve a balance between the reliable results and computing times. In this inflation step, hard contact between stent inner surface and balloon outer surface was defined with a friction coefficient of 0.25. This is different from the friction coefficient of 0.8 used for the contact between the outer surface of stent and rigid plates during crimping. The chosen value (0.25) was based on literature (Ju et al. 2008), as physical measurement was not available. This value was able to prevent the sliding of the stent in the longitudinal direction (Schiavone et al. 2014, 2016). Following stent expansion, a deflation step was performed to allow free recoil of the stent.

### Post-processing of the results

In this study, the whole process of stent crimping and expansion was simulated using Abaqus/Explicit. In all simulations, the internal and kinetic energies were monitored to make sure that the kinetic energy was always less than 5% of internal energy. Automatic time increment was applied, and the time increment was in the order of $10^{-7}$ s throughout the analysis.

In FE studies, the von Mises stress has been widely used to assess the stress state on stent, plaque and artery (Ju et al. 2008; Imani et al. 2015; Eshghi et al. 2011). In this study, the von Mises stress contour plot was used to reveal the stress distribution of stent. Stent recoiling, radial stiffness and strength were calculated to quantify the stent performance during crimping and expansion processes.

The elastic recoil is determined by the deformation behaviour of the material and the specific stent structure (Lanzer 2007), so stent recoil can be used to evaluate the stent design. Specifically, recoiling effect is calculated by (ASTM F2079 2017):

$$ \text{Stent Recoil }(\%) = (d_{0}-d_{1})/d_{0} \times 100\%, $$

where $d_{0}$ and $d_{1}$ represent the diameters in the middle of stent at peak pressure and after balloon deflation, respectively.

Radial stiffness and radial strength can be calculated from the radial loading curves produced in re-crimping procedure. Generally, a radial loading curve is a plot of radial pressure as a function of diametric deformation of a stent. The radial pressure is expressed as a radial force divided by the instantaneous stent cylindrical area (ASTM F3067 2014):

$$ A=\pi DL, $$

where $D$ is instantaneous stent outer diameter, and $L$ is the original stent length.

Radial stiffness is the slope of the steepest initial linear portion of the radial loading curve. Radial strength is defined as the radial pressure at which the stent experiences irrecoverable deformation, and it is determined by the 0.1 mm offset line which is parallel to the slope of initial linear portion.

## Results and discussions

Four commercially available bioresorbable stents were simulated in order to compare the performances of different stent designs. Residual stress caused by crimping was considered in all simulations. The effect of lading rate on stent deformation was investigated for the Elixir stent.

### Stent crimping

**Fig. 4:**
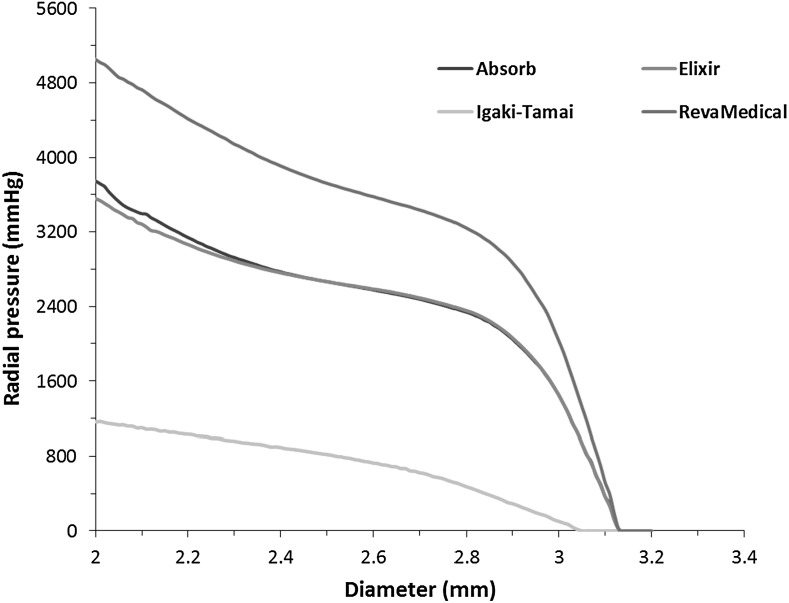
Stent diameter change against pressure during crimping process

Figure 4 shows the diameter change against radial pressure for four stents in the crimping procedure. It can be seen that in all four stents, the Igaki–Tamai stent required the least radial pressure for crimping, because it has the smallest surface area and stent length in the design. Also the Igaki–Tamai stent has a larger spacing of axial struts and a larger amplitude of U-bends, leading to much reduced rigidity. The Absorb and Elixir stents showed very similar performance in the crimping procedure due to similar designs. The RevaMedical stent required the most radial pressure for crimping, suggesting its high radial stiffness and strength.

**Fig. 5:**
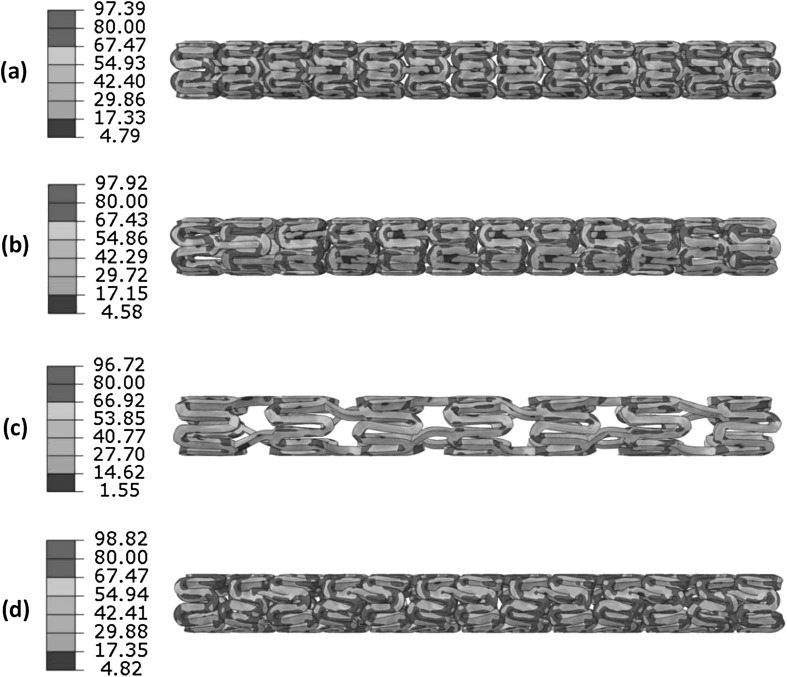
The von Mises stress (MPa) contour plot for (**a**) Absorb, (**b**) Elixir, (**c**) Igaki–Tamai and (**d**) RevaMedical stents in fully crimped configuration

All four stents were squeezed to a diameter of around 1.2 mm at the end of crimping procedure. Figure 5 shows the von Mises stress distributions for four stents in fully crimped configuration. In all cases, maximum stresses occurred at U-bends where the red colour was mostly located. It is clear that the Igaki–Tamai stent had less high stress level area when compared to the other three stents, because it experienced less deformation with the longer U-bend and axial strut spacing in design. The maximum von Mises stresses on the stents after crimping had a very similar magnitude, which was 97.39 MPa, 97.92 MPa, 96.72 MPa and 98.82 MPa for the Absorb, Elixir, Igaki–Tamai and RevaMedical stent, respectively.

**Fig. 6:**
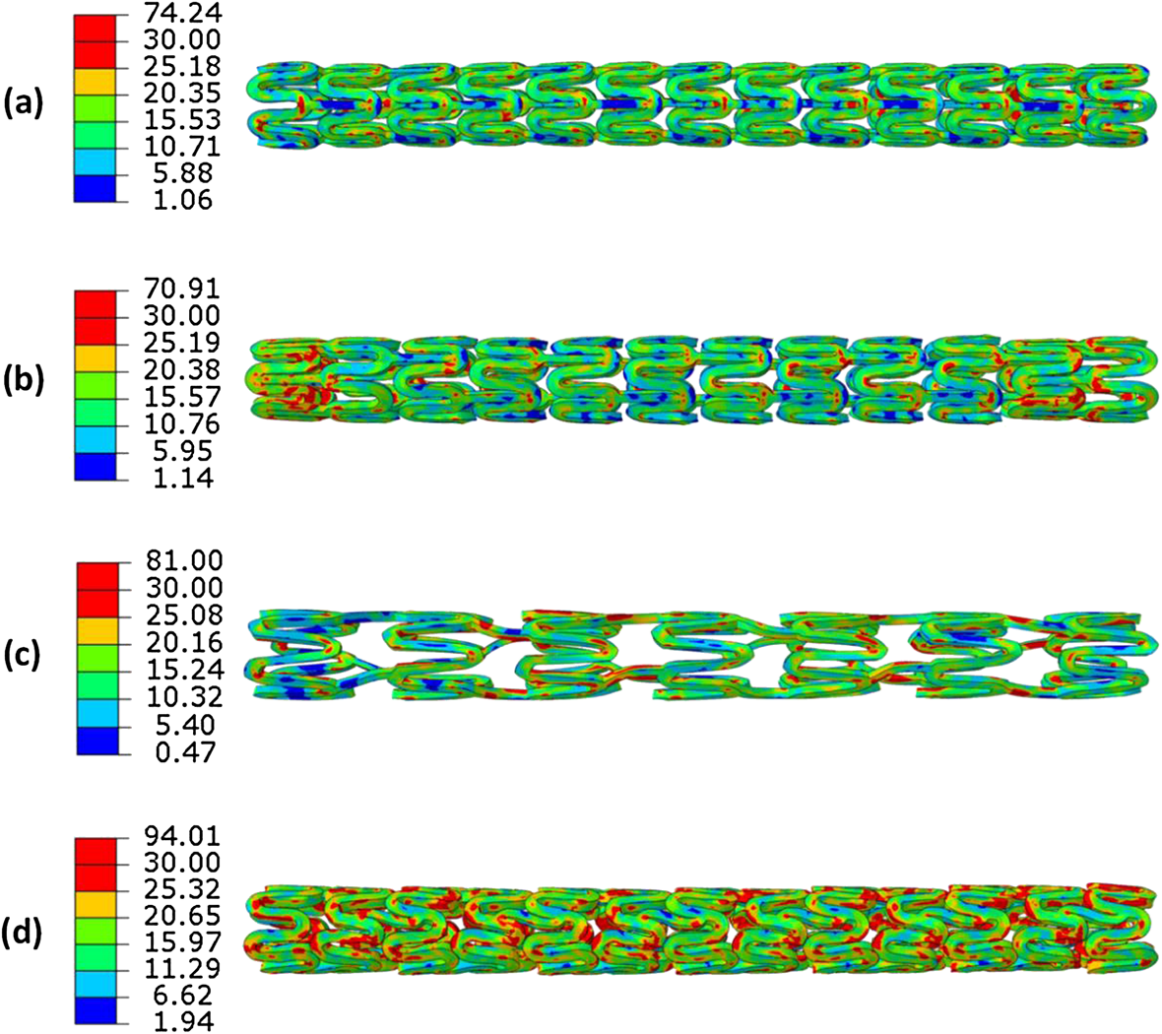
The von Mises stress (MPa) contour plot (**a**) Absorb, (**b**) Elixir, (**c**) Igaki–Tamai and (**d**) RevaMedical stents after spring back

**Fig. 7:**
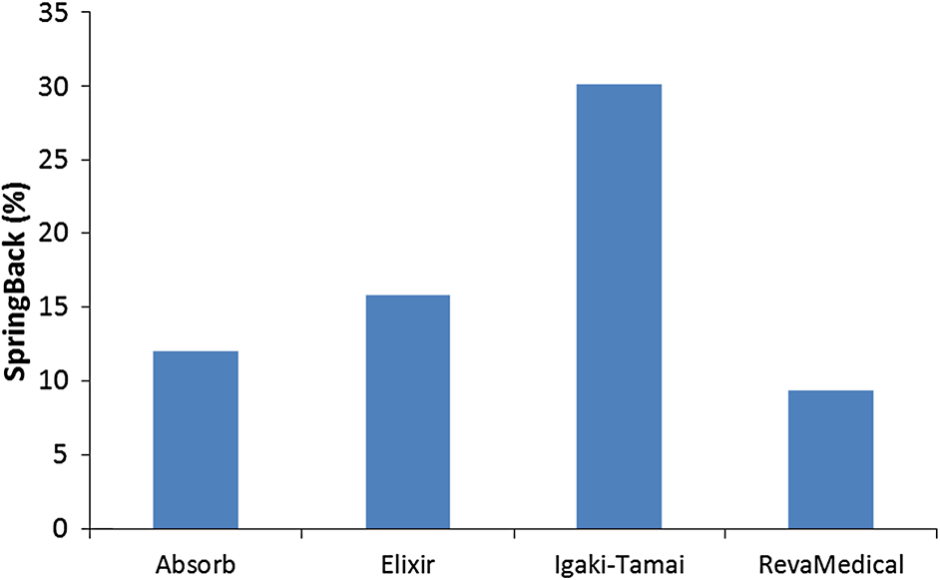
Spring-back effects for four stents after releasing rigid plates

Figure 6 gives the von Mises stress distributions for the four stents after spring back. These stresses, called residual stress caused by crimping, all reduced due to the elastic spring-back effects. The residual stresses turned out to be 74.24 MPa for the Absorb stent, 70.91 MPa for the Elixir stent, 81.00 MPa for the Igaki–Tamai stent and 94.01 MPa for the RevaMedical stent. The spring-back effects for four stents were calculated and shown in Fig. 7. The Igaki–Tamai stent had the most spring back (30%), which was significantly higher than the other three stents. The spring back was 12% for the Absorb stent, 15% for the Elixir stent, and 10% for the RevaMedical stent.

The material properties were defined exactly the same for all four bioresorbable polymeric stents, but they have different mechanical performances during crimping processes, due to their individual designs. The Absorb and Elixir stent have the same design of U-bend (e.g., radius and amplitude), so the von Mises stress distributions are very similar. However, some difference was found regarding residual stresses and spring-back effect for these two stents. The Elixir stent sprung back more than the Absorb stent, because the Elixir stent has a larger axial strut spacing that gives it more freedom to recover. In addition, the Elixir stent has two axial struts between each two ring units but the Absorb stent has three, so the Elixir stent experienced more spring back and the loss of more stress during spring back. Similarly, the Igaki–Tamai stent presented the biggest spring-back effect due to its large U-bend and axial strut spacing. The RevaMedical stent has the same U-bend design as Absorb and Elixir stents, but it presented less spring-back effect. It is probably due to the different strut thickness. The RevaMedical stent has a larger strut thickness (200 μm) than those of Absorb and Elixir stents (150 μm).

### Stent expansion

**Fig. 8:**
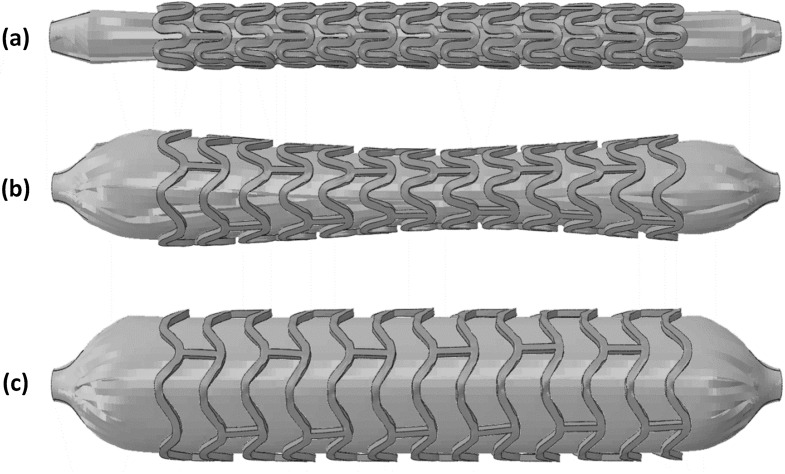
Configurations of the Absorb stent and balloon at an inflating pressure of (**a**) 0 MPa, (**b**) 0.5 MPa, and (**c**) 1.4 MPa

Figure 8 presents the configurations of Absorb stent and balloon at different intervals during the inflation process. At zero pressure, the Absorb stent has the crimped shape (Fig. 8a). When the pressure increased to 0.5 MPa, the stent turned out to be in a typical dogbone shape. It is because the tri-folded balloon expanded first from both free ends (lack of resistance from stent), which caused faster expansion of the stent at the two ends than in the middle. The tri-folded balloon was fully inflated at the pressure of 1.4 MPa, and the dogbone shape disappeared for the stent. This phenomenon was also reported in the literature (Debusschere et al. 2015).

**Fig. 9:**
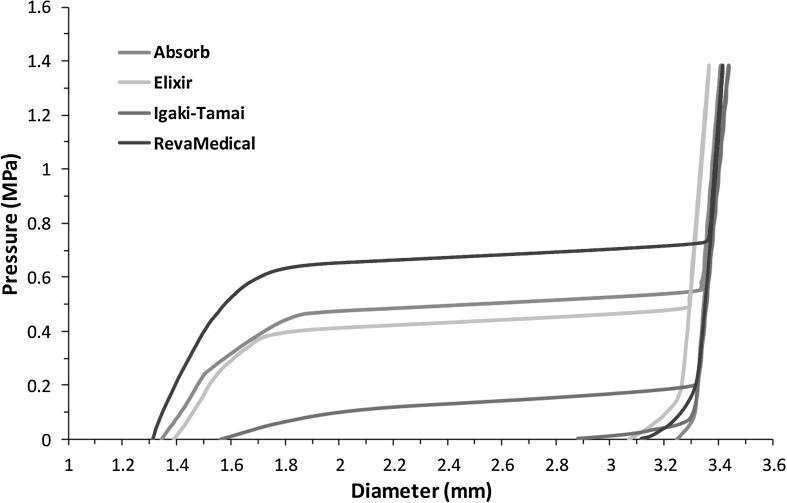
Stent diameter change against pressure for the four stents during expansion process

Figure 9 shows the stent diameter change against pressure of all four stents during their expansion processes. Under the same inflating pressure rate, these four stents had different expanding rate and achieved a different diameter after balloon deflation. Obviously, the Igaki–Tamai stent expanded with the fastest rate and reached saturation earlier than the other three stents. The RevaMedical stent experienced rapid expansion at a pressure of 0.7 MPa, while the Absorb and Elixir stents required lower pressure (0.5 MPa and 0.4 MPa) to reach the state of rapid expansion. The diameters at peak pressure were very close for all four stents, which are within a range of $3.3\sim 3.4~\text{mm}$. The final diameter achieved after balloon deflation was 3.25 mm for the Absorb stent, 3.07 mm for the Elixir stent, 2.88 mm for the Igaki–Tamai stent and 3.12 mm for the RevaMedical stent.

**Fig. 10:**
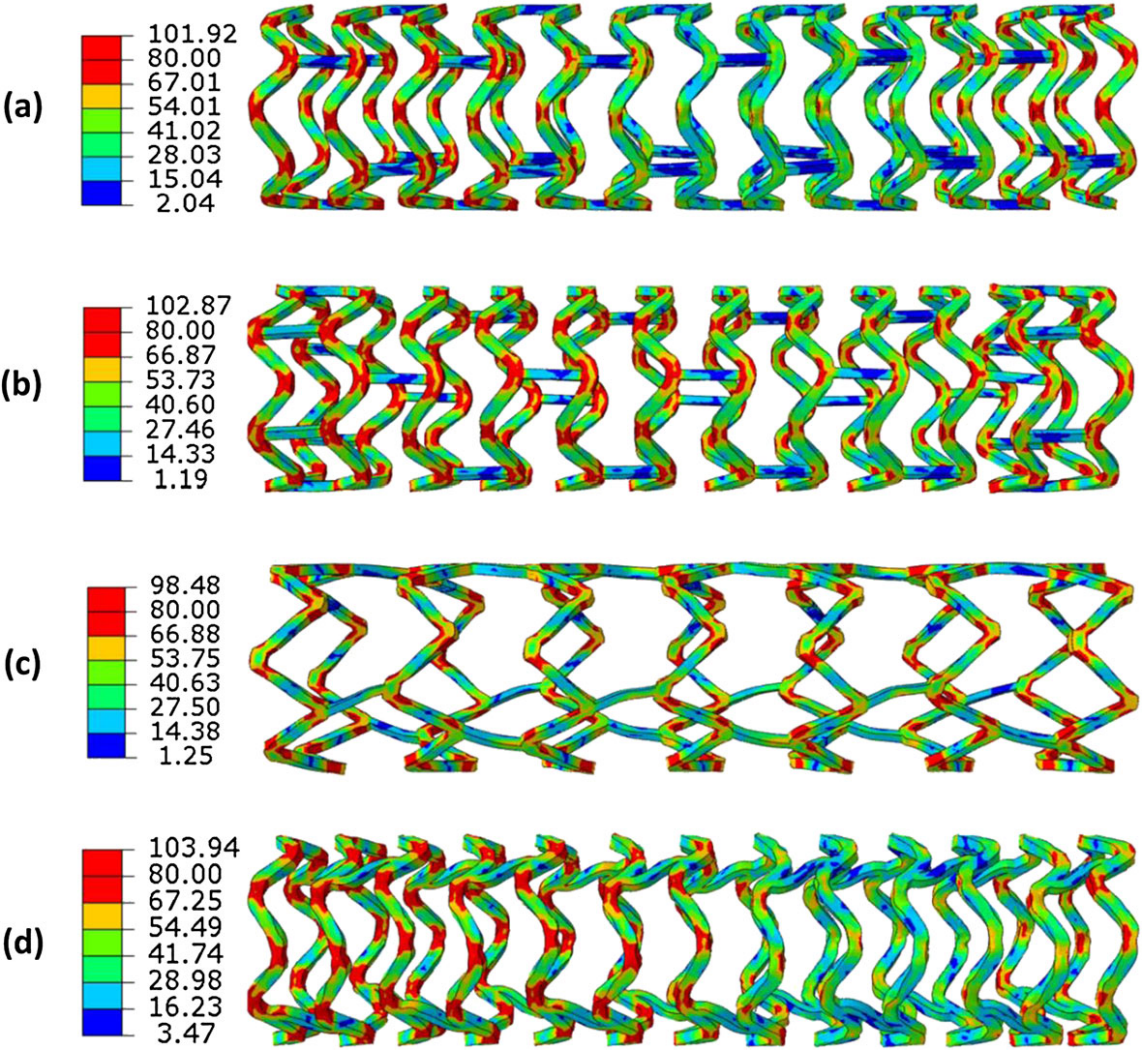
The von Mises stress (MPa) contour plot for (**a**) Absorb, (**b**) Elixir, (**c**) Igaki–Tamai and (**d**) RevaMedical stents at peak pressure

Figure 10 shows the von Mises stress distribution on stents at peak pressure. The red areas indicate that the stresses are higher than 80 MPa. All four stents had uniformly expanded shapes. Similar to the crimping result, in all cases, stress concentrations were found at the inner and outer corners of U-bends. The maximum stresses were 101.92 MPa for the Absorb stent, 102.87 MPa for the Elixir stent, 98.48 MPa for the Igaki–Tamai stent and 103.94 MPa for the RevaMedical stent.

**Fig. 11:**
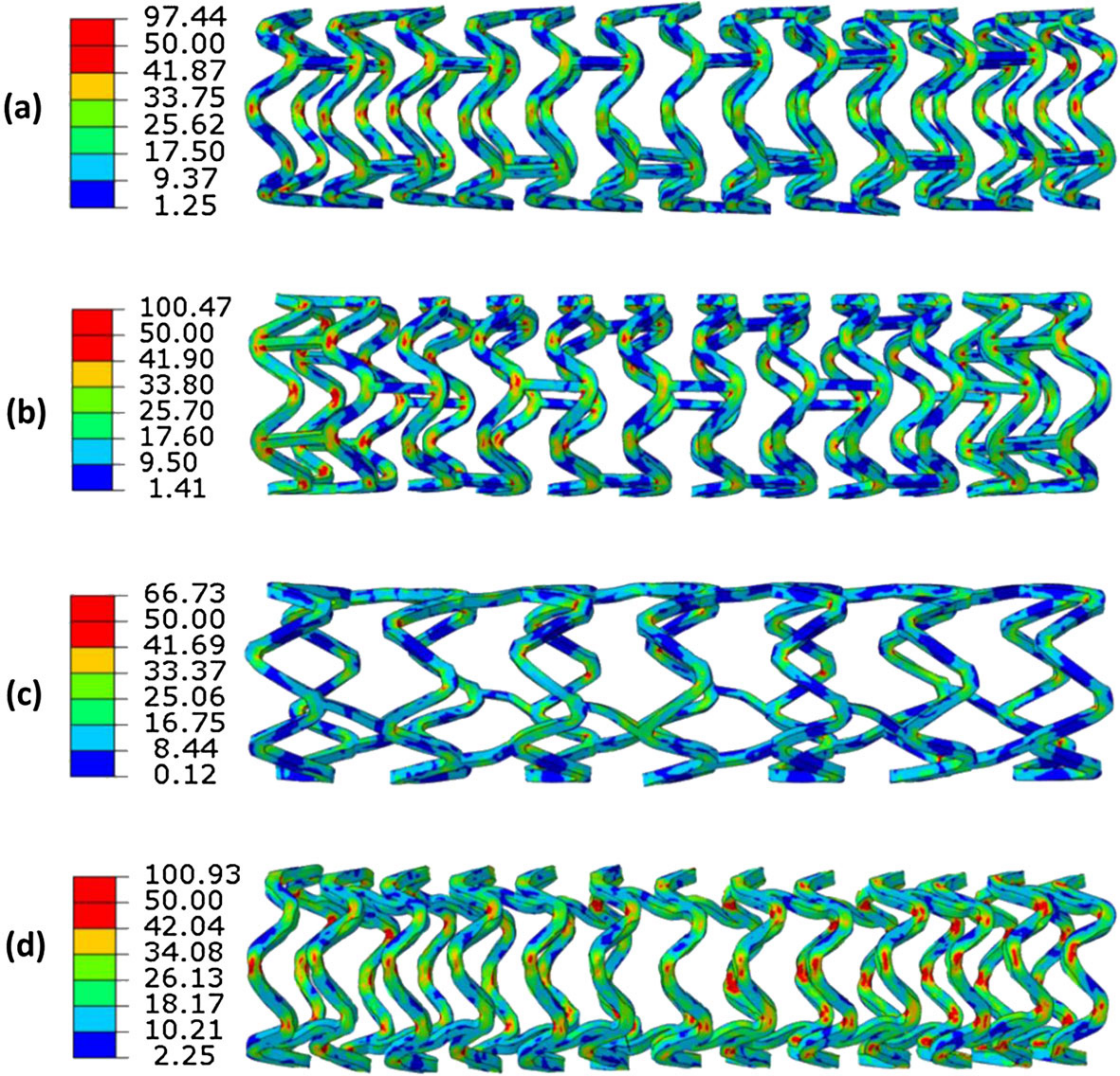
The von Mises stress (MPa) contour plot for (**a**) Absorb, (**b**) Elixir, (**c**) Igaki–Tamai and (**d**) RevaMedica stents after balloon deflation

**Fig. 12:**
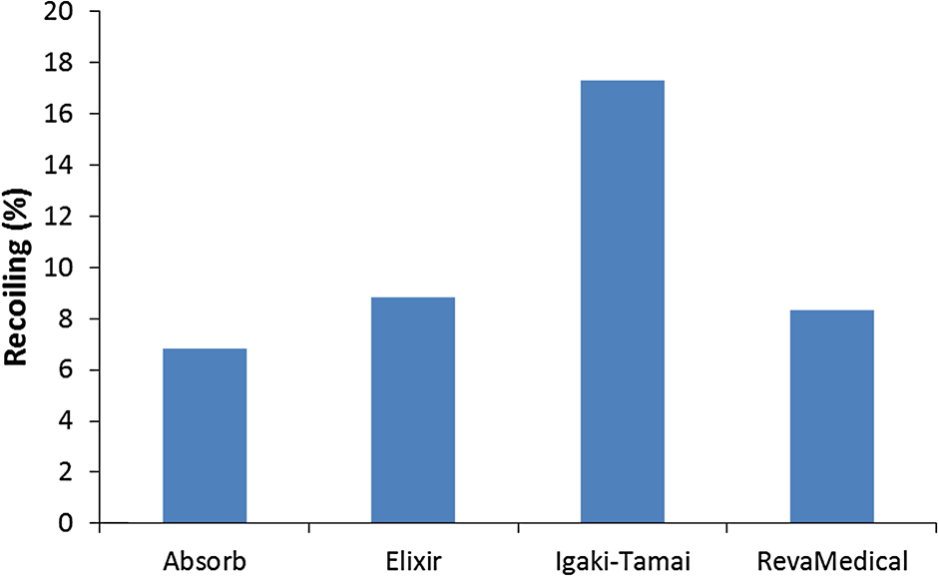
Recoiling effects for four stents after balloon deflation

Figure 11 presents the stress distribution on stents after deflation. There was a stress loss for all four stents. The maximum stress magnitudes turned out to be 97.44 MPa for the Absorb stent, 100.47 MPa for the Elixir stent, 66.73 MPa for the Igaki–Tamai stent, and 100.93 MPa for the RevaMedical stent. Although the maximum stress magnitude did not change much in most of cases, high stress regions (highlighted in red colour) were significantly reduced on stents due to the recoiling effect. The recoiling effects for all four stents are shown in Fig. 12. The Igaki–Tamai stent had the most stress loss during deflation, and thus experienced the most recoiling effect (17%). In addition, the recoiling was 6.8%, 8.82% and 8.34% for Absorb, Elixir and RevaMedical stents, respectively.

### Residual stresses caused by crimping

**Fig. 13:**
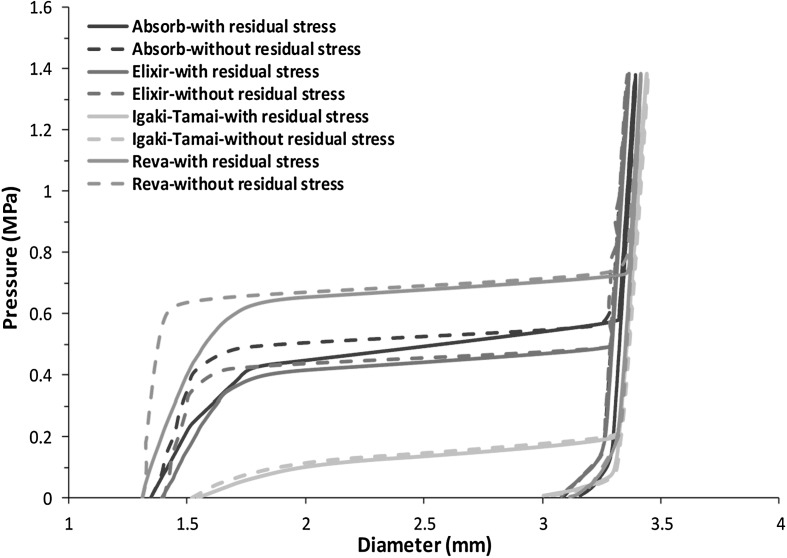
Diameter change against balloon pressure obtained from simulations with and without residual stress on stents

Significant amount of residual stress was generated during the crimping procedure as shown in Fig. 6, so it is important to study how these residual stresses affected the stent expansion behaviour. The stent expansion behaviour presented above was obtained by taking residual stress into account. Simulations of stent expansion were also carried out by excluding the residual stress state. Figure 13 presented the stent diameter change against pressure during stent expansion process, with and without considering residual stress. In the inflation step, it showed that the presence of residual stress affected the expansion of stents at very early stage only. However, the pressure at which stent expansion reached saturation was 0.7 MPa, 0.5 MPa, 0.4 MPa and 0.1 MPa for RevaMedical, Elixir, Absorb and Igaki–Tamai stents, respectively, which were comparable to those obtained in the simulations with the consideration of residual stress. The stent diameters achieved at peak pressure did not change greatly either. For instance, the Absorb stent reached a diameter of 3.39 mm at peak pressure in the simulation considering residual stress, and achieved a diameter of 3.36 mm in the simulation without considering residual stress. In the deflation step, similar behaviour was observed for stent diameter change obtained in simulations with or without considering residual stresses. Moreover, the final diameters obtained after balloon deflation were also very similar for these four stents. For instance, the Absorb stent achieved final diameter of 3.160 mm and 3.164 mm in simulations with and without considering residual stress, respectively.

**Fig. 14:**
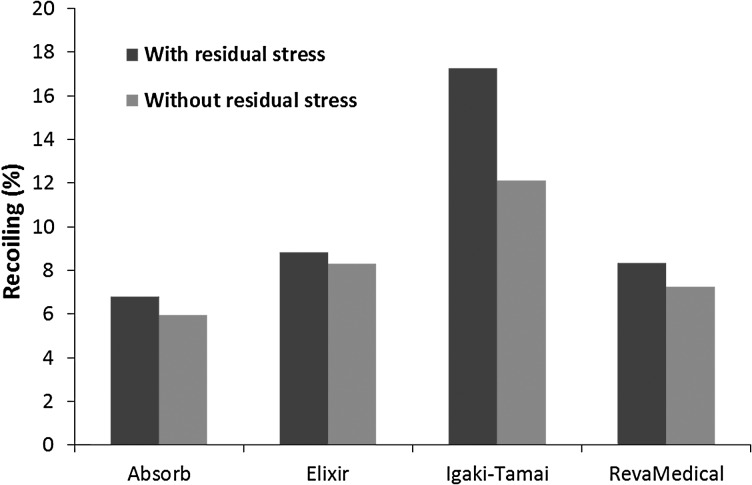
Recoiling effect obtained from simulations with and without considering residual stress

Figure 14 shows the recoiling effect results obtained from simulations with and without considering residual stress for these four stents. A slight difference was found for the Absorb, Elixir and RevaMedical stents. The recoiling for the Igaki–Tamai stent was 17% in the simulation when considering residual stress, but reduced to 12% when the residual stress was not considered. This suggested that the effect of residual stress should be considered in the computational study of expansion behaviour for stent with specific design.

### Radial stiffness and strength

**Fig. 15:**
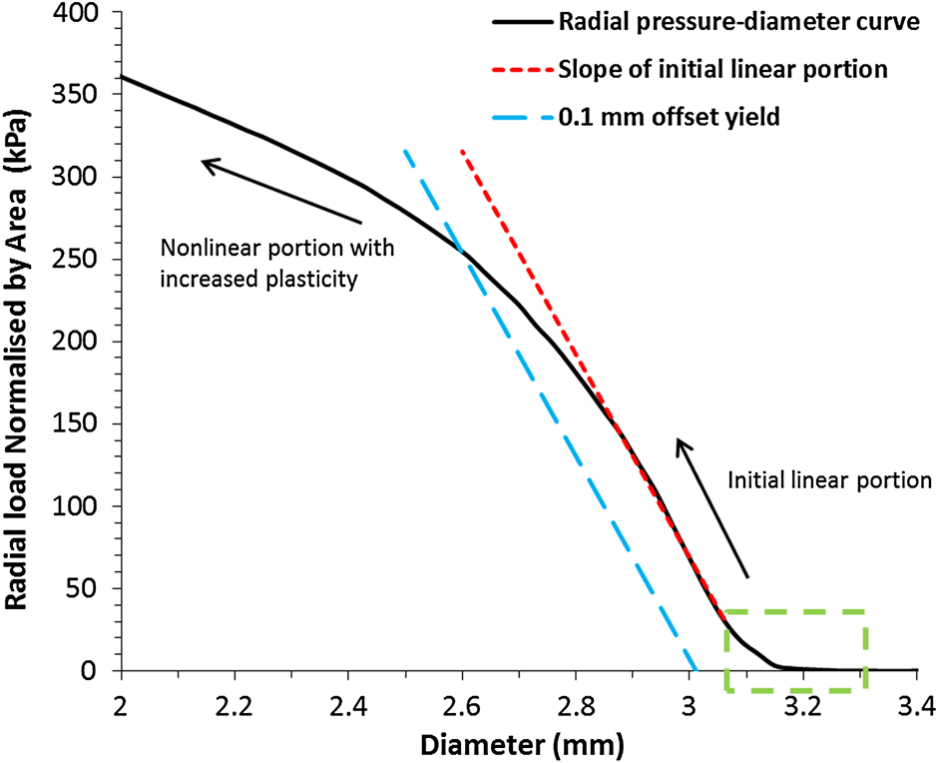
Radial loading plot of Absorb stent during re-crimping process

Figure 15 shows the radial pressure-diameter plot for the Absorb stent during re-crimping process. The behaviour can be divided into two regions, the initial linear behaviour and the follow-on nonlinear behaviour with increased plasticity. For initial linear region, there was a slight nonlinear portion before the rigid plates started to contact the outer surface of stent, as shown in green box. After the full contact was established, the curve became approximately linear. In the linear portion of the curve, the stent underwent mainly elastic deformation. The slope of the linear portion determines the radial stiffness of the stent, which is 616 kPa/mm for the Absorb stent. With the increase of plastic deformation, the curve tended to become nonlinear. During this region, the radial pressure increased relatively slowly with the decrease of stent diameter. The interception between the 0.1 mm offset line and the pressure-diameter curve determines the radial strength of the stent, which was 250 kPa in this case.

Similarly, the radial stiffness and radial strength were also calculated for the other three stents (Table 1). Obviously, the RevaMedical stent presented the highest radial stiffness (708 kPa/mm) and strength (318 kPa). The Elixir stent, with very similar design to Absorb, presented a slightly lower radial stiffness (538 kPa/mm) and strength (218 kPa). The calculated radial stiffness and strength were 132 kPa/mm and 73 kPa for the Igaki–Tamai stent, which are much lower when compared to the other three stents. This suggests that stent design plays an important role in controlling the radial stiffness and strength of stents.

**Table 1:** Radial stiffness and radial strength for four stents

Stent	Absorb	Elixir	Igaki–Tamai	RevaMedical
Radial stiffness (kPa/mm)	616	538	132	708
Radial strength (kPa)	250	218	73	318

In this study, the Absorb, Elixir and RevaMedical stents showed comparable radial stiffness to that reported by Pauck and Reddy (2015). In their work, the radial stiffness was approximately 500 kPa/mm for a typical polymeric stent ($E = 1.8~\text{GPa}$ and $\text{yield stress} = 45~\text{MPa}$), and increased to 1300 kPa/mm when the material modulus was increased to 3.6 GPa. This indicates that material property affects the radial properties of stents. In addition, the RevaMedical stent showed higher radial stiffness and strength than the Absorb and Elixir stents, despite the same U-bend design, suggesting that the strut thickness also greatly affects the radial property of stent.

### Effect of loading rate

**Fig. 16:**
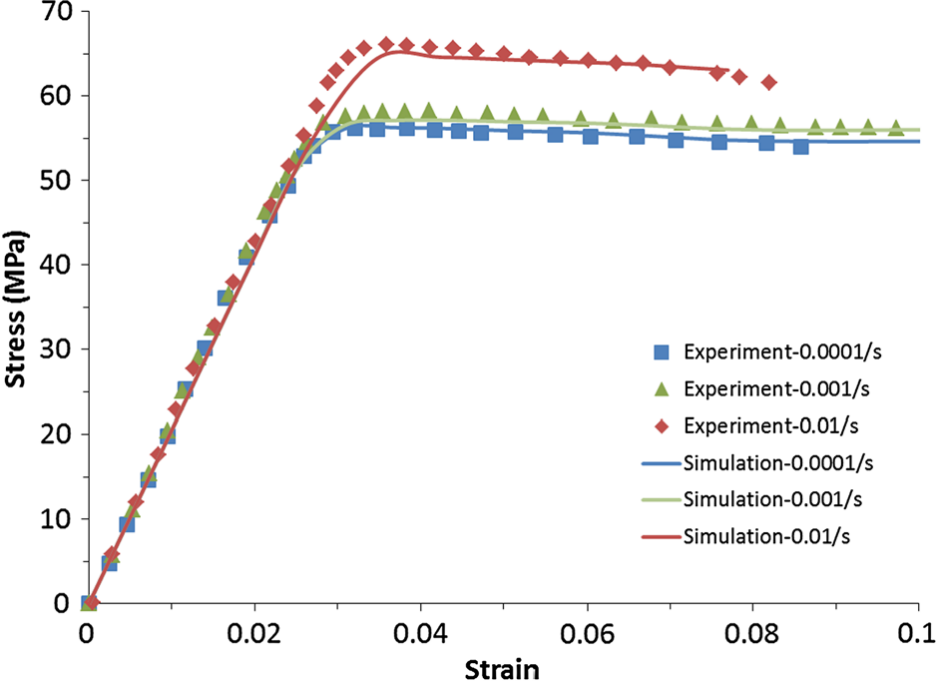
Experimental and simulated stress-strain behaviours of blended PLA/PBS (70/30) for different strain rates

Strain rate has an effect on the stress-strain behaviour of polymers, demonstrating the time-dependent deformation nature of the materials, i.e., an increase of stress with the increase of strain rate. This time-dependent or viscous behaviour needs to be considered properly in the design and manufacturing of components and devices made of polymers, including computational modelling. In this section, an elastic–plastic model with rate dependency, available in ABAQUS (2016), was used to simulate expansion of Elixir stent under three different loading rates (1.4, 14 and 140 MPa/s). Again, isotropic hardening was implemented by the description of yield stress as a function of plastic strain. Strain rate dependence was defined by inputting yield-stress ratios as a function of equivalent plastic strain rate. The yield-stress ratio is the ratio of the yield stress at nonzero strain rate to the static yield stress. As shown in Fig. 16, the time-dependent stress-strain behaviour of a PLA (poly-lactic-acid)/PBS (poly-butylene-succinate) blend (with a 70/30 weight ratio), measured experimentally in Qiu et al. (2016), was captured very well by the model.

**Fig. 17:**
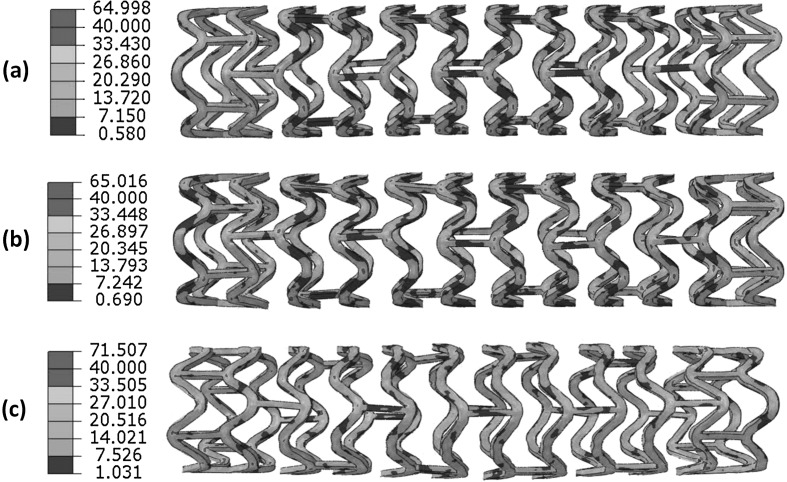
The von Mises stress (MPa) contour plot for the Elixir stent after expansion, with a loading rate of (**a**) 1.4 MPa/s, (**b**) 14 MPa/s and (**c**) 140 MPa/s

**Fig. 18:**
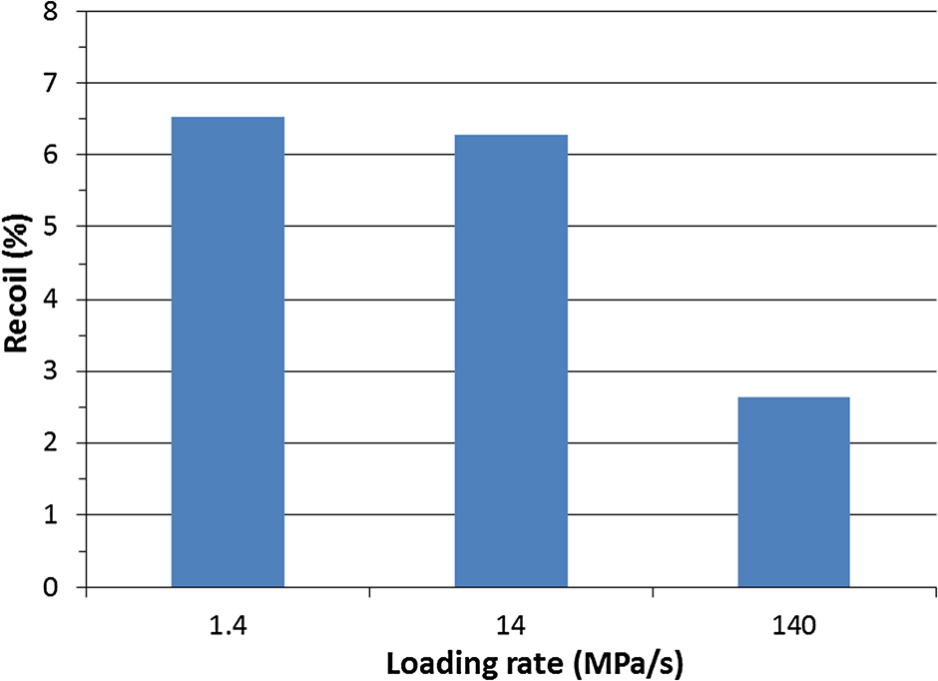
Recoiling effect of the Elixir stent for different loading rates

The von Mises stress contour plot is given in Fig. 17 for the stent after expansion under three different loading rates (i.e., 1.4 MPa/s, 14 MPa/s and 140 MPa/s). Stress variation was clearly observed for the three loading rates, in terms of both distribution and magnitude. Figure 18 shows a significant decrease of stent recoiling when the loading rate was increased to 140 MPa/s. These results demonstrate that stent exhibits different mechanical behaviour under different loading rates, in terms of stress level and recoiling effects. With the increase of loading rate, stent experienced more expansion, with less recoiling effect but higher stress. These results suggest that loading rate should be considered as a factor in the clinical practice of stent implantation. It should be noted that the material used for the rate-effect study in this section is different from the PLLA material simulated in previous sections. This is because the rate-dependent stress-strain behaviour is not available for the PLLA material. However, the general conclusion is believed to be valid.

## Conclusions

In this study, stent crimping and expansion were modelled for four commercial bioresorbable polymeric stents, i.e., Absorb, Elixir, Igaki–Tamai and RevaMedical stent, using finite element method. Due to individual designs, these four stents experienced different mechanical performances during crimping and expansion processes. Major conclusions are as follows:

(I) The Absorb, Elixir and RevaMedical stents showed very similar mechanical performance, in terms of stress distribution, spring-back/recoiling effects as well as radial stiffness and strength, due to the same U-bend design.
(II) The Igaki–Tamai stent, with large U-bend and axial strut spacing, turned out to be less resistant to elastic recoil and recovery.
(III) Residual stress caused by crimping was found to reduce the expansion rate of stents, and it also affected the recoiling.
(IV) The radial stiffness and radial strength of stent were influenced by the structural designs (e.g., radius and amplitude of U-bend, axial strut spacing and strut thickness) and material properties.
(V) With the increase of loading rate, stent experienced more expansion with less recoiling effect but higher stress, suggesting that loading rate should be considered as a factor in stent implantation.

## References

[CR1] ABAQUS, 6.14, Dassault Systemes Simulia Corp., Providence, USA (2016)

[CR2] Abizaid A., Costa R.A., Schofer J., Ormiston J., Maeng M., Witzenbichler B., Botelho R.V., Costa J.R., Chamié D., Abizaid A.S., Castro J.P. (2016). Serial multimodality imaging and 2-year clinical outcomes of the novel DESolve novolimus-eluting bioresorbable coronary scaffold system for the treatment of single de novo coronary lesions. JACC, Cardiovasc. Interv..

[CR3] ASTM F2079 Standard Test Method for Measuring Intrinsic Elastic Recoil of Balloon Expandable Stents (2017)

[CR4] ASTM F3067 Guide for Radial Loading of Balloon Expandable and Self Expanding Vascular Stents (2014)

[CR5] De Beule M., Mortier P., Carlier S.G., Verhegghe B., Van Impe R., Verdonck P. (2008). Realistic finite element-based stent design: the impact of balloon folding. J. Biomech..

[CR6] Debusschere N., Segers P., Dubruel P., Verhegghe B., De Beule M. (2015). A finite element strategy to investigate the free expansion behaviour of a biodegradable polymeric stent. J. Biomech..

[CR7] Eshghi N., Hojjati M.H., Imani M., Goudarzi A.M. (2011). Finite element analysis of mechanical behaviors of coronary stent. Proc. Eng..

[CR8] Garcia-Garcia H.M., Serruys P.W., Campos C.M., Muramatsu T., Nakatani S., Zhang Y.J., Onuma Y., Stone G.W. (2014). Assessing bioresorbable coronary devices: methods and parameters. JACC, Cardiovasc. Interv..

[CR9] Gervaso F., Capelli C., Petrini L., Lattanzio S., Di Virgilio L., Migliavacca F. (2008). On the effects of different strategies in modelling balloon-expandable stenting by means of finite element method. J. Biomech..

[CR10] Grabow N., Bünger C.M., Sternberg K., Mews S., Schmohl K., Schmitz K.P. (2007). Mechanical properties of a biodegradable balloon-expandable stent from poly (L-lactide) for peripheral vascular applications. J. Med. Devices.

[CR11] Imani S.M., Goudarzi A.M., Valipour P., Barzegar M., Mahdinejad J., Ghasemi S.E. (2015). Application of finite element method to comparing the NIR stent with the multi-link stent for narrowings in coronary arteries. Acta Mech. Solida Sin..

[CR12] Ju F., Xia Z., Sasaki K. (2008). On the finite element modelling of balloon-expandable stents. J. Mech. Behav. Biomed. Mater..

[CR13] Lanzer P. (2007). Mastering Endovascular Techniques: A Guide to Excellence.

[CR14] Migliavacca F., Petrini L., Montanari V., Quagliana I., Auricchio F., Dubini G. (2005). A predictive study of the mechanical behaviour of coronary stents by computer modelling. Med. Eng. Phys..

[CR15] Nishio S., Kosuga K., Igaki K., Okada M., Kyo E., Tsuji T., Takeuchi E., Inuzuka Y., Takeda S., Hata T., Takeuchi Y. (2012). Long-term ($> 10$ years) clinical outcomes of first-in-human biodegradable poly-l-lactic acid coronary stents Igaki–Tamai stents. Circulation.

[CR16] Pauck R.G., Reddy B.D. (2015). Computational analysis of the radial mechanical performance of PLLA coronary artery stents. Med. Eng. Phys..

[CR17] Qiu T., Song M., Zhao L.G. (2016). Testing, characterization and modelling of mechanical behaviour of poly (lactic-acid) and poly (butylene succinate) blends. Mech. Adv. Mater. Mod. Process..

[CR18] Schiavone A., Zhao L.G., Abdel-Wahab A.A. (2014). Effects of material, coating, design and plaque composition on stent deployment inside a stenotic artery–finite element simulation. Mater. Sci. Eng. C.

[CR19] Schiavone A., Abunassar C., Hossainy S., Zhao L.G. (2016). Computational analysis of mechanical stress-strain interaction of a bioresorbable scaffold with blood vessel. J. Biomech..

[CR20] Schiavone A., Qiu T., Zhao L.G. (2017). Crimping and deployment of metallic and polymeric stents–finite element modelling. Vessel Plus.

[CR21] Serruys P.W., Ormiston J.A., Onuma Y., Regar E., Gonzalo N., Garcia-Garcia H.M., Nieman K., Bruining N., Dorange C., Miquel-Hébert K., Veldhof S. (2009). A bioabsorbable everolimus-eluting coronary stent system (ABSORB): 2-year outcomes and results from multiple imaging methods. Lancet.

[CR22] Tamai H., Igaki K., Kyo E., Kosuga K., Kawashima A., Matsui S., Komori H., Tsuji T., Motohara S., Uehata H. (2000). Initial and 6-month results of biodegradable poly-l-lactic acid coronary stents in humans. Circulation.

[CR23] Varcoe R.L., Schouten O., Thomas S.D., Lennox A.F. (2016). Experience with the absorb everolimus-eluting bioresorbable vascular scaffold in arteries below the knee: 12-month clinical and imaging outcomes. JACC, Cardiovasc. Interv..

[CR24] Wang Q., Fang G., Zhao Y., Wang G., Cai T. (2017). Computational and experimental investigation into mechanical performances of Poly-L-Lactide Acid (PLLA) coronary stents. J. Mech. Behav. Biomed. Mater..

[CR25] Welch T.R., Eberhart R.C., Chuong C.J. (2009). The influence of thermal treatment on the mechanical characteristics of a PLLA coiled stent. J. Biomed. Mater. Res., Part B, Appl. Biomater..

[CR26] Welch T.R., Eberhart R.C., Reddy S.V., Wang J., Nugent A., Forbess J. (2013). Novel bioresorbable stent design and fabrication: congenital heart disease applications. Cardiovasc. Eng. Technol..

[CR27] Wiebe J., Nef H.M., Hamm C.W. (2014). Current status of bioresorbable scaffolds in the treatment of coronary artery disease. J. Am. Coll. Cardiol..

